# *Goniozus omanensis* (Hymenoptera: Bethylidae) an important parasitoid of the lesser date moth *Batrachedra amydraula* Meyrick (Lepidoptera: Batrachedridae) in Oman

**DOI:** 10.1371/journal.pone.0223761

**Published:** 2019-12-11

**Authors:** A. Polaszek, T. Almandhari, L. Fusu, S. A. H. Al-Khatri, S. Al Naabi, R. H. Al Shidi, S. Russell, I. C. W. Hardy

**Affiliations:** 1 Dept of Life Sciences, Natural History Museum, London, England, United Kingdom; 2 School of Biosciences, University of Nottingham, Sutton Bonington Campus, England, United Kingdom; 3 Plant Protection Research Centre, Ministry of Agriculture and Fisheries, Muscat, Sultanate of Oman; 4 Faculty of Biology, 'Al. I. Cuza' University, Iasi, Romania; 5 Core Research Laboratories, Natural History Museum, London, England, United Kingdom; Institute of Plant Physiology and Ecology Shanghai Institutes for Biological Sciences, CHINA

## Abstract

A new species of bethylid parasitoid wasp, *Goniozus omanensis* Polaszek **sp. n.**, is described based on morphology and DNA sequence data. The species is currently known only from the lesser date moth *Batrachedra amydraula*, a pest of economic importance, but can be reared on two factitious host species. *G*. *omanensis* is compared with *G*. *swirskiana*, known from the same host in Israel. We summarise current knowledge of *G*. *omanensis* life-history, and its potential as an agent of biological pest control.

## Introduction

Date palm cultivation is widespread in many countries with hot and dry climates. The lesser date moth *Batrachedra amydraula* Meyrick (Lepidoptera: Batachedridae) is one of the major pests of date palms in the Arabian Peninsula and neighbouring countries, including Egypt, Iran, Iraq, Israel and Libya. Unripe fruits are attacked early in the growing season by larvae that bore into them. Fruits attacked initially remain attached to their stalks by larval silk, but eventually dry and drop, resulting in considerable yield loss, which can reach 70–80% [1–5] in Oman, *B*. *amydraula* produces three generations between February and June, after which larvae remain dormant before pupating the following year and emerging as adults [6].

Natural enemies of *B*. *amydraula* have been surveyed in Egypt [7], Iraq [8] and Oman [9], and at least one species of *Goniozus* has been reared from this pest. *Goniozus* is a genus of small wasps in the family Bethylidae; the member species are usually gregarious ectoparasitoids of lepidopteran larvae, especially stem borers, leafminers, leafrollers and fruit borers [10, 11]. *Goniozus swirskiana* (Argaman) was described from *B*. *amydraula* hosts in Israel [12]. More recently, a *Goniozus* species common on *B*. *amydraula* in Oman [9, 13] has been referred to informally as “*Goniozus omani*” [14] Specimens from Oman were sent to the first author in 2017 and examined morphologically, including the preparation of male genitalia, critical for recognising *Goniozus* at species-level. The conclusion based on morphology alone was that these specimens belong to an undescribed species, and therefore were not *G*. *swirskiana*. The same specimens then underwent a DNA extraction protocol that leaves the sclerotized structures intact, and the resulting DNA sequence data supported the initial conclusion that the specimens belonged to an undescribed species. Here we describe this new species, as *Goniozus omanensis* Polaszek, both to facilitate future identification, and to provide the formal nomenclature essential to support further work using this parasitoid. We also provide a summary of its currently known biology, and areas for further research.

## Materials and methods

### Specimen depositories: Abbreviations

NHMUK: Natural History Museum, London UK.

ONHM: Oman Natural History Museum, Muscat, Oman.

USNM: United States National Museum, Washington D.C., USA.

### Morphological study

Specimens were obtained from the mass-rearing culture (T.A., unpublished data; see also Supporting Information 1). Card-mounted specimens were observed with a Leitz binocular microscope at magnifications ranging from 10 to 40×. Side mounted structures were observed with a Leitz Dialux 20 EB compound microscope at magnifications ranging between 40x and 400×. Several specimens were gold-palladium coated and photographed with a Zeiss Ultra Plus field emission Scanning Electron Microscope at magnifications between 300× and 900×. Images were generated as follows: Light microscope images: Canon DSLR with 10× Mitutoyo objective, processed with HeliconFocus stacking software; Compound microscope images (slide-mounted structures): Leitz Dialux 20EB compound microscope using Nomarski Differential Interference Contrast illumination, photographed with MicroPublisher 5.0 RTV camera; scanned sections stacked and combined using Synoptics AutoMontage^®^ software; Scanning electron micrographs: Zeiss Ultra Plus field emission Scanning Electron Microscope. All final image editing with Adobe Photoshop CC^®^. Morphological terminology largely follows Azevedo *et al* [15] with the exception of that for male genitalia, which follows Williams [16] and some other terms for which the equivalents from Azevedo *et al* [15] are provided.

The holotype of *G*. *omanensis* is deposited at NHMUK, paratypes in NHMUK, ONHM and USNM.

### DNA sequencing

Four *Goniozus omanensis* individuals from Oman (3 females, 1 male), and two *Goniozus* females from Iraq *ex Batrachedra amydraula*, were subjected to “non-destructive” DNA extraction. Genomic DNA was extracted using the protocol described in Polaszek *et al*.[17] and Cruaud *et al*.[18], which leaves the sclerotized parts of the specimen intact. Specimens were then critical point dried and card-mounted, with selected individuals then dissected and mounted in Canada balsam on microscope slides, and others gold-palladium coated for SEM examination.

To generate CO1 sequences, the standard “barcode” forward primer LCO1490 [19] was paired with the reverse primer C1-N-2329 (aka K525) [20]. The resulting amplicon is longer than the standard “barcode” region by about 160 bp. The PCR cycle for the 5’ end of the CO1 consisted of an initial denaturation step of 94°C for 2 min, followed by 40 cycles of 94°C for 30 s, 40°C for 60 s and 72°C for 30 s, and a final extension step of 10 min at 72°C. For 28S the conditions were similar, except for annealing at 55°C for 30 s.

The 28S D2 fragment was amplified with the primers D23F (5′-GAG AGT TCA AGA GTA CGT G-3′) [21] and D2R (aka 28S-Rev) (5′-TTG GTC CGT GTT TCA AGA CGG-3′) [22]. All reactions were carried out in 25 μl reaction volume containing 5 μl of template DNA, 2.5 μl of 10× PCR buffer, 0.75 μl of 50 mM MgCl2, 0.2 μl dNTPs solution (25 mM each), 1.25 μl of each primer (10 μM), 0.3 μl Taq polymerase (5u/μl Biotaq, Bioline), and PCR grade water to final volume.

Both DNA strands were sequenced at the Natural History Museum Life Sciences DNA Sequencing Facility (London) using the same primers used for the PCR. Forward and reverse sequences were assembled and edited as described in Fusu and Ribes [23].

### Phylogenetic analyses

For the phylogenetic analyses we assembled a dataset by first using a BLAST search on GenBank to retrieve the most similar sequences, and then searching and downloading all the available *Goniozus* sequences (CO1 sequences identical to others from GenBank were removed). We did not use an outgroup, the trees being rooted at midpoint, since the phylogenetic relationships within Bethylidae are still not confidently resolved, for example, based on molecular data, *Odontepyris* Kieffer renders *Goniozus* paraphyletic [24]. DNA sequences were aligned using ClustalW [25] and CO1 sequences were further translated into the amino acid sequence to check for the presence of stop codons. Genetic distances were calculated using the *p*-distance. All of the above was carried out using Mega 7 [26].

The CO1 alignment was partitioned by codon position in Mesquite v3.10 [27] and the best partitioned scheme and substitution models were then identified using PartitionFinder 2 [28] by restricting the search to those models available in MrBayes v3 [29] and using linked branch lengths plus the greedy algorithm [30]. The 28S D2 alignment was treated as a single partition due to the lack of an appropriate secondary structure model and the best substitution model identified using jModeltest v2.1.10 [31]. Phylogenetic trees were inferred using maximum likelihood in RAxML v8.2.12 [32] by using the GTR+G substitution model (the only available model) with 1000 bootstrap pseudo replicates and Bayesian inference in MrBayes v3.2.6 with the best partitioning scheme and substitution models identified as described above. For the Bayesian inference two parallel analyses, each with four chains, were run for 10 000 000 generations, with trees and lnLs sampled every 100 generations. Convergence was assessed by examining the trace files in Tracer v1.7. [33]. The first 25% of the sampled trees were discarded as burn-in. The trees were examined and modified for presentation in FigTree v1.4.2 (A. Rambaut, https://github.com/rambaut/figtree/releases). Since the results of the two inference methods were very similar, posterior probabilities were plotted on the maximum likelihood trees in Adobe Illustrator^®^.

### Nomenclatural acts

The electronic edition of this article conforms to the requirements of the amended International Code of Zoological Nomenclature, and hence the new names contained herein are available under that Code from the electronic edition of this article. This published work and the nomenclatural acts it contains have been registered in ZooBank, the online registration system for the ICZN. The ZooBank LSIDs (Life Science Identifiers) can be resolved and the associated information viewed through any standard web browser by appending the LSID to the prefix "http://zoobank.org/". The LSID for this publication is: urn:lsid:zoobank.org:pub:CE45C3B6-038B-4107-AADB-9EA5BA8254BA. The electronic edition of this work was published in a journal with an ISSN, and has been archived and is available from the following digital repositories: PubMed Central, LOCKSS.

## Description

### *Goniozus omanensis* Polaszek sp. n.

urn:lsid:zoobank.org:act:148B35EB-4B8B-44E4-AC56-1F4BC5F70508

Figs 1–10

**Fig 1:**
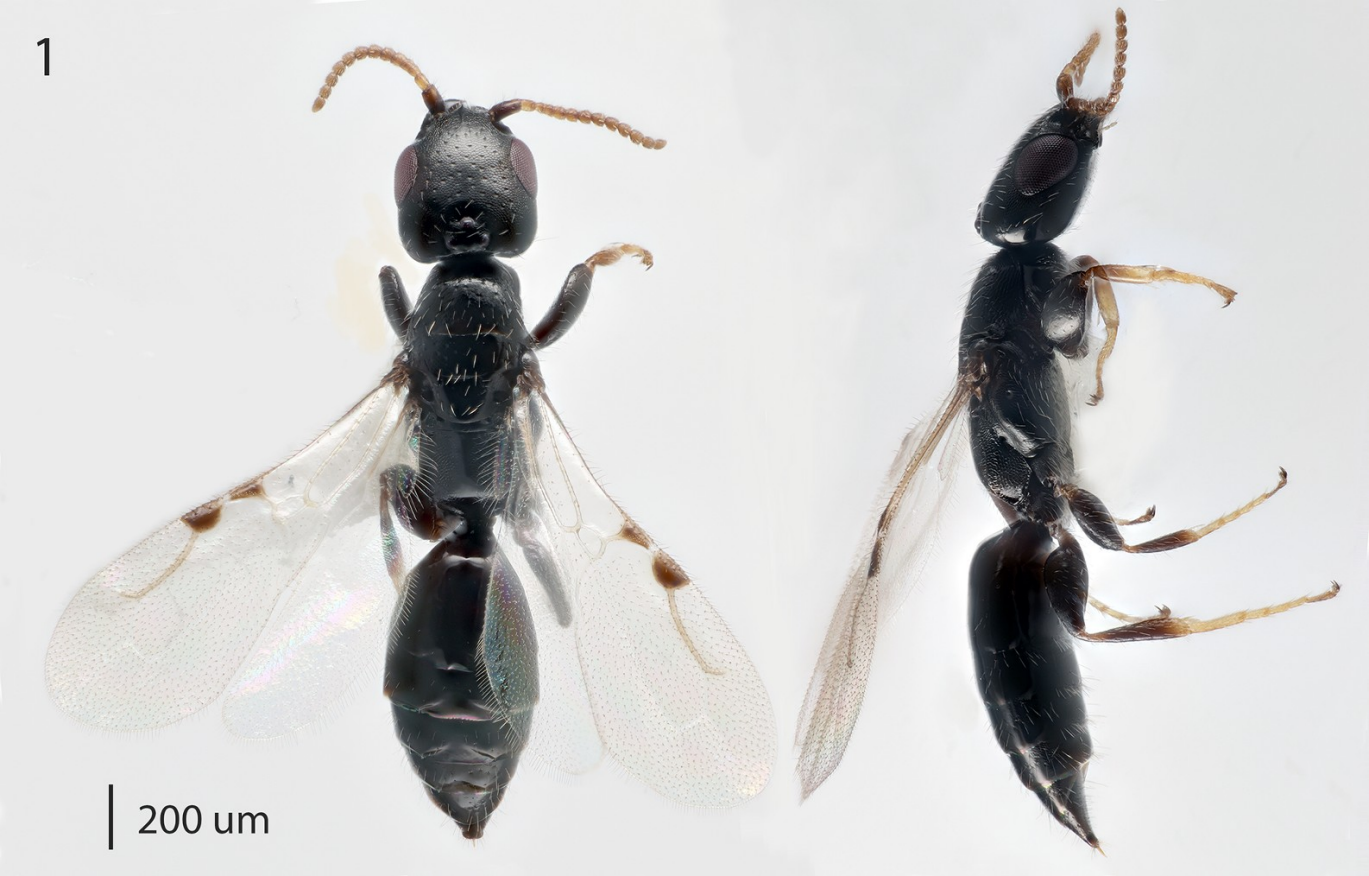
*Goniozus omanensis* Polaszek female holotype. Left: dorsal habitus; Right: lateral habitus.

**Fig 2:**
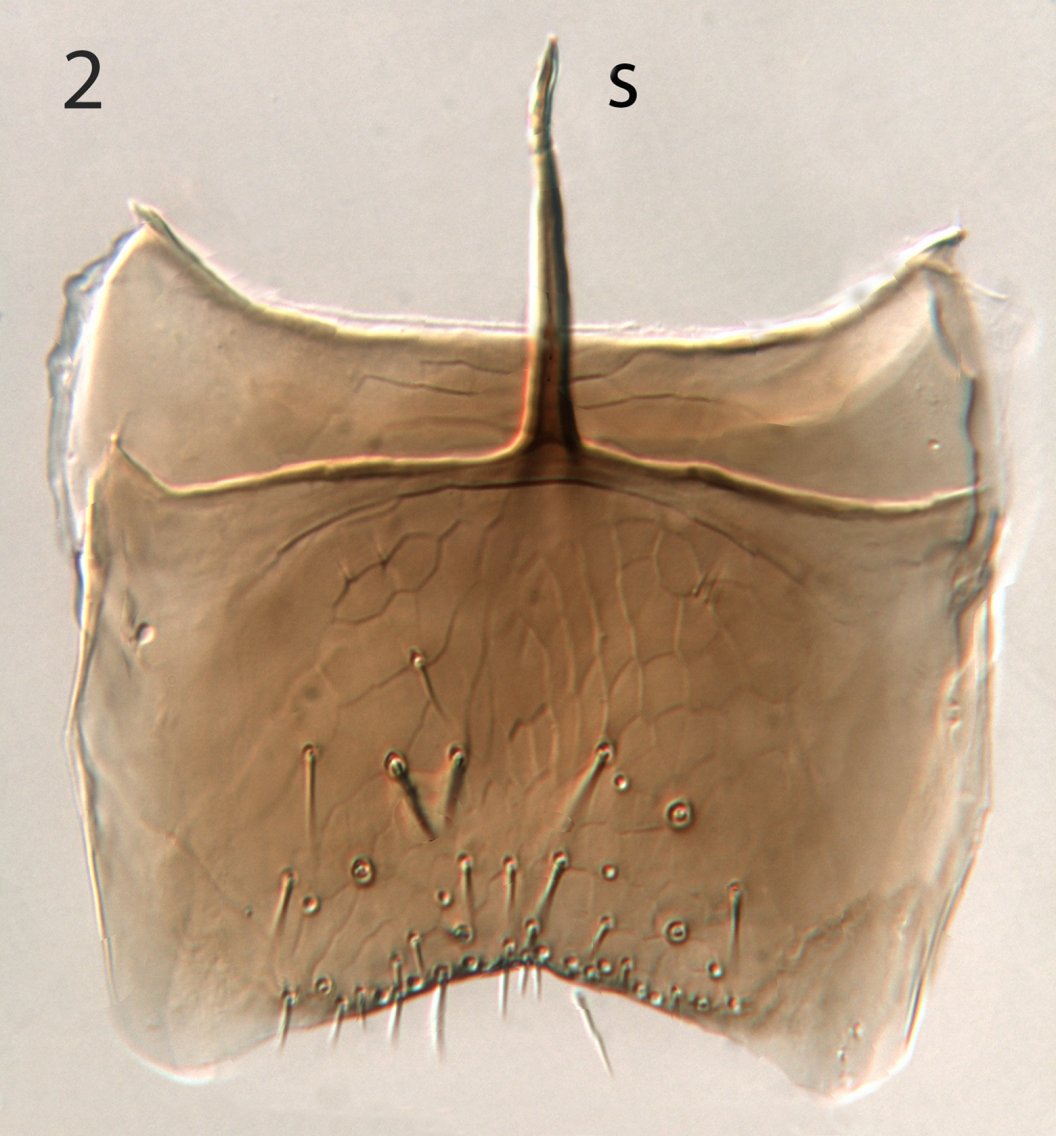
*G*. *omanensis* male paratype, genitalia; subgenital plate + 8^th^ sternite. S = speculum.

**Fig 3:**
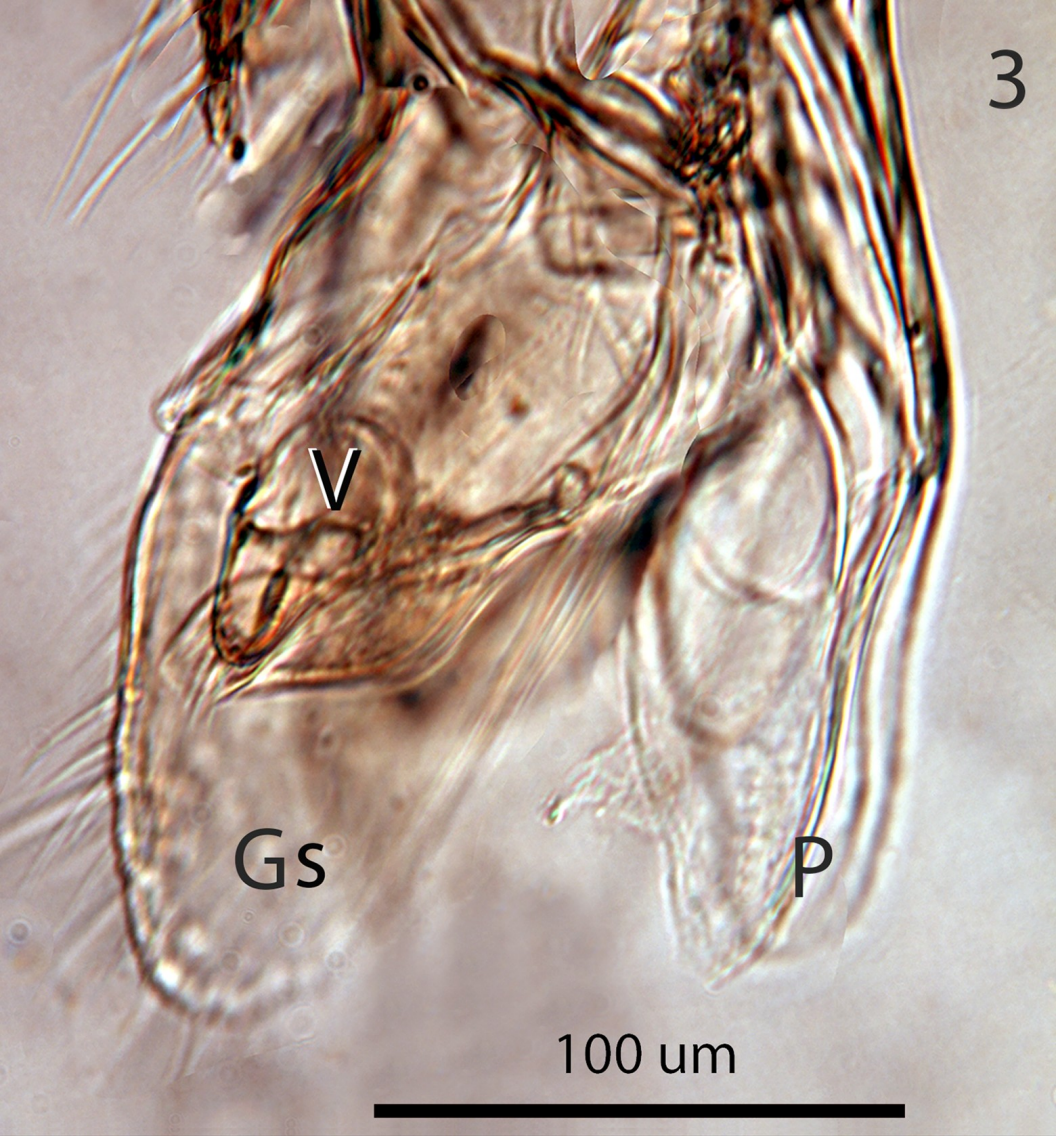
*G*. *omanensis* male paratype, lateral apex of genital capsule. Gs = gonostylus; P = penis valves.

**Fig 4:**
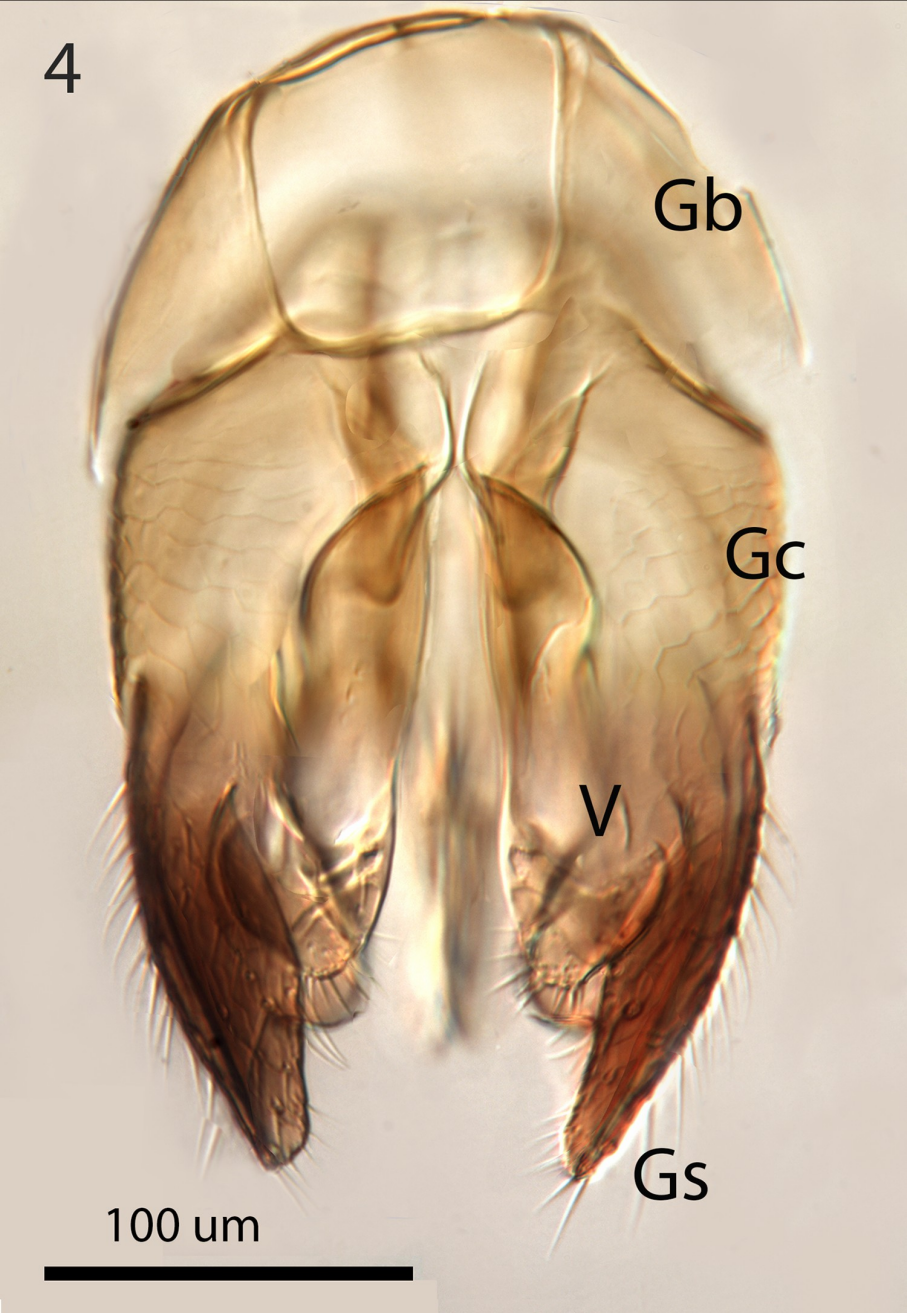
*G*. *omanensis* male paratype, dorsal genital capsule. Gb = gonobase; Gc = gonocoxite; Gs = gonostylus.

**Fig 5:**
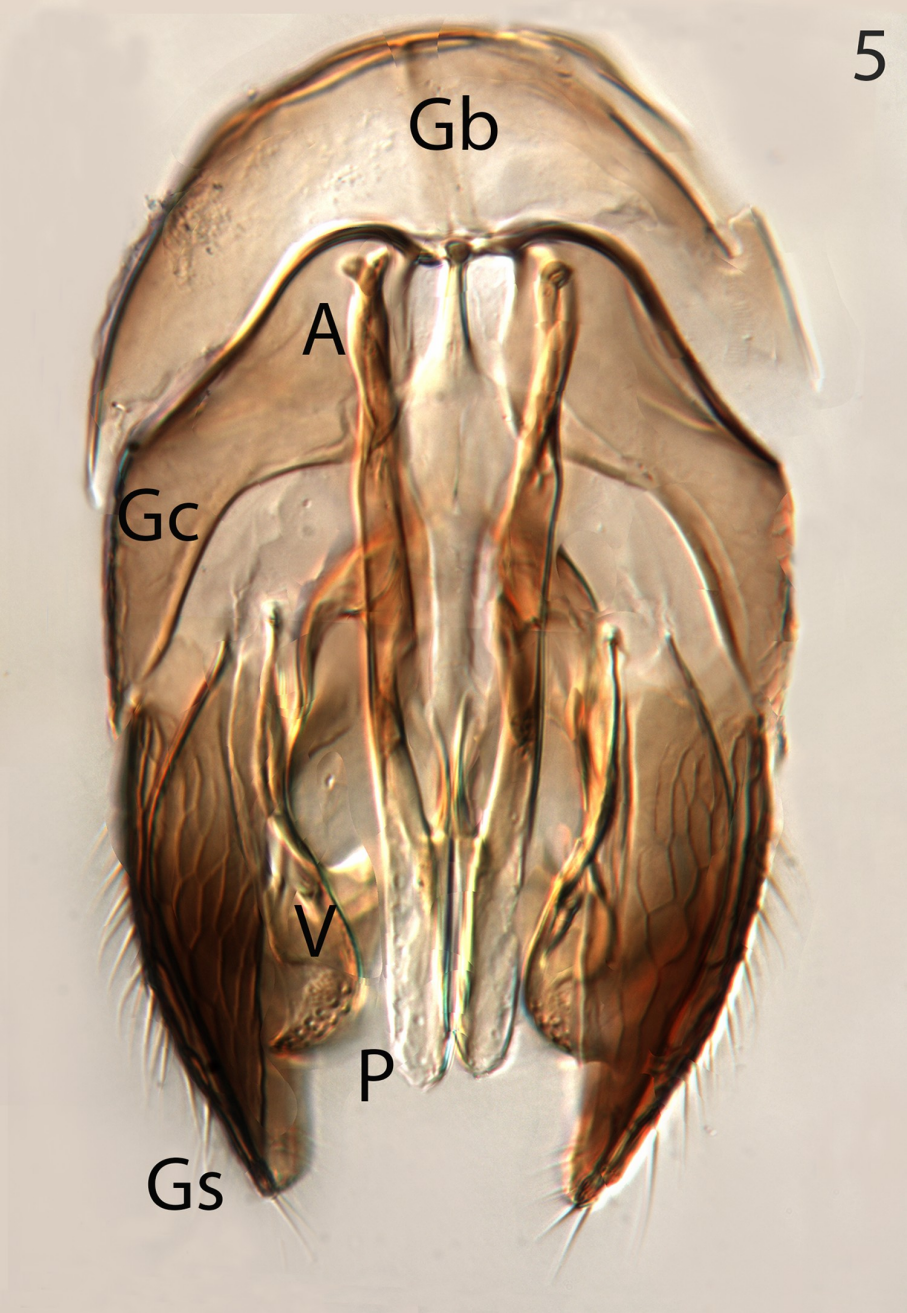
*G*. *omanensis* male paratype, ventral genital capsule. Gb = gonobase; A = apodemes of penis valves; Gc = gonocoxite; Gs = gonostylus.

**Fig 6:**
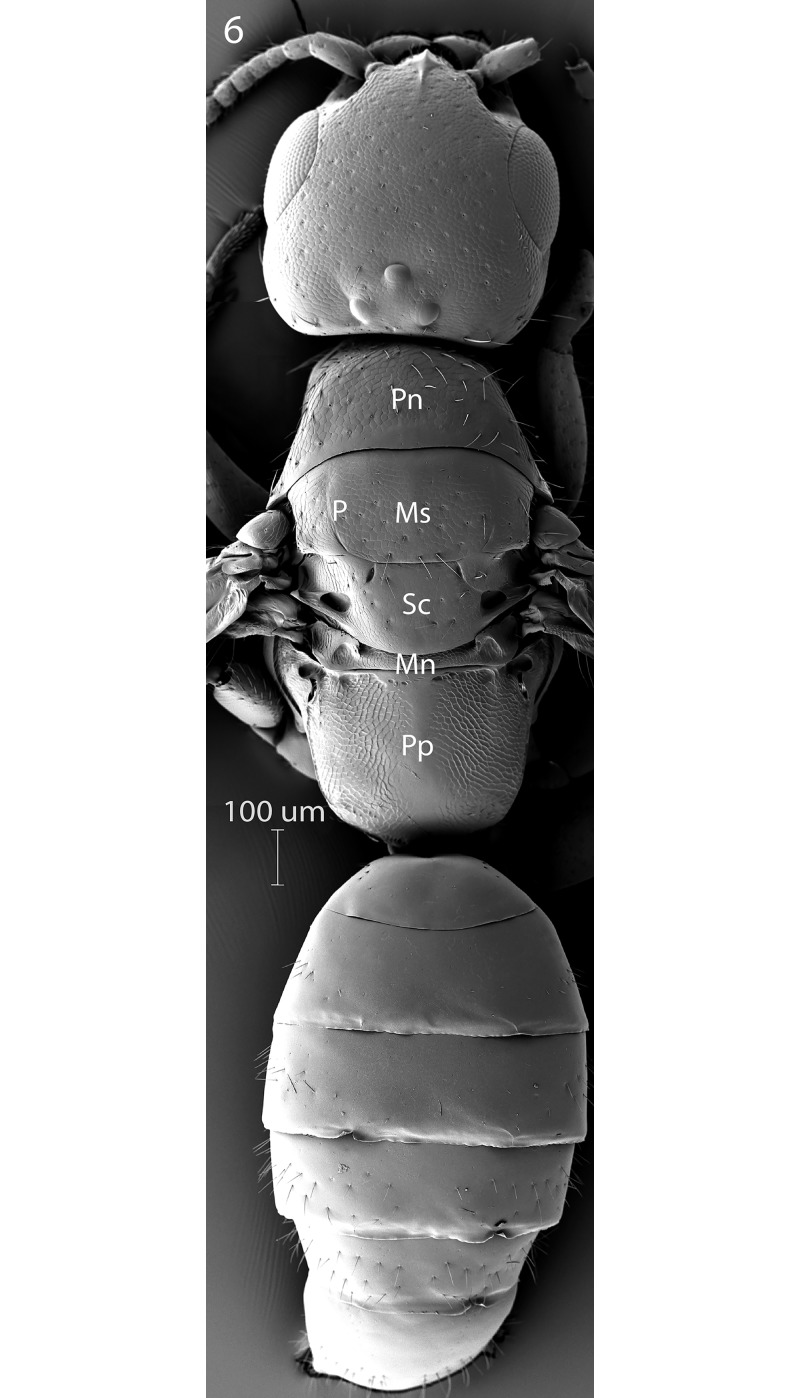
*G*. *omanensis* female paratype, SEM, dorsal habitus. Pn = pronotum; Ms = mesoscutum; P = parapsidal line; Sc = scutellum; Mn = metanotum; Pp = propodeum.

**Fig 7:**
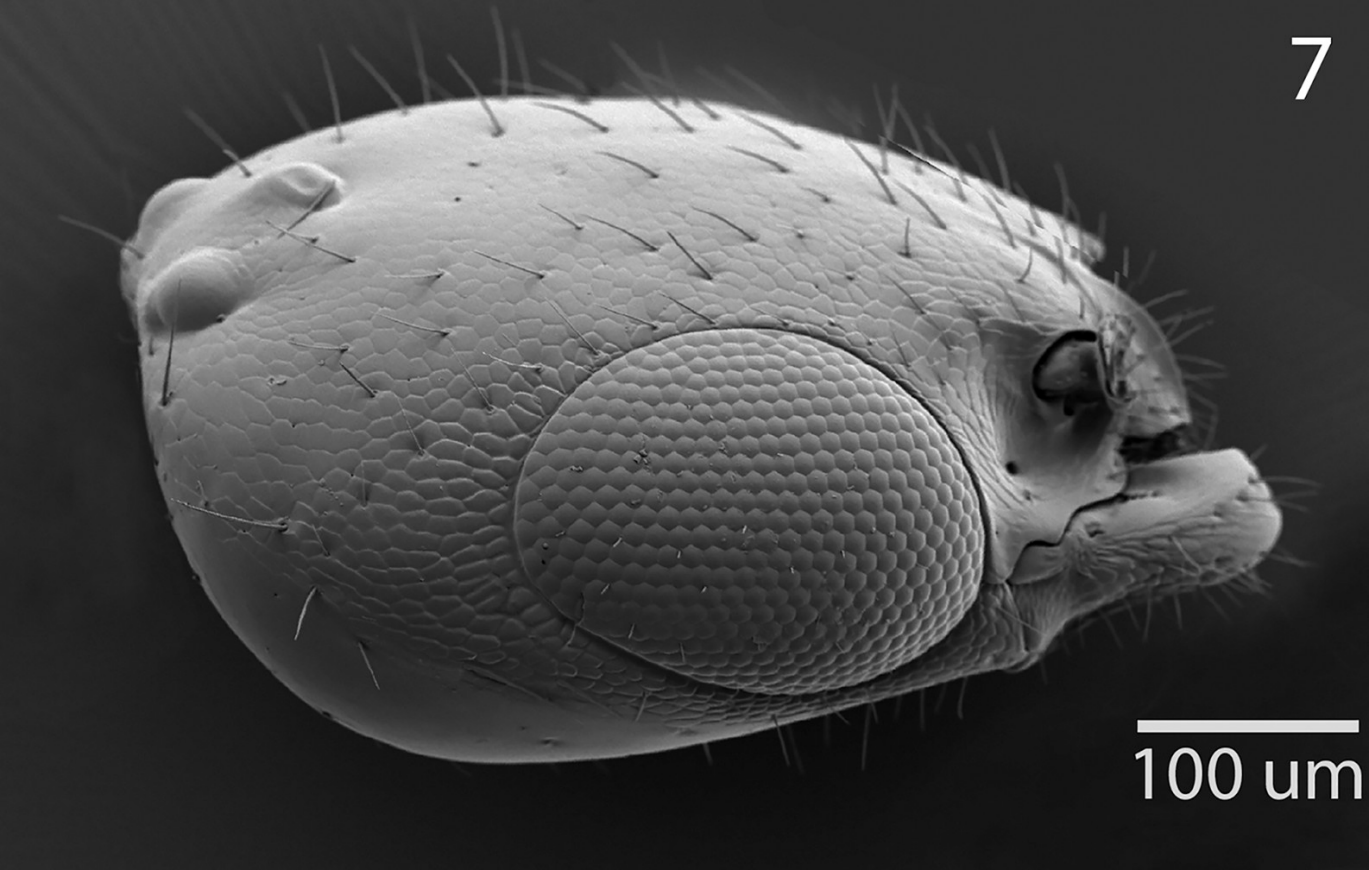
*G*. *omanensis* female paratype, SEM Head, lateral.

**Fig 8:**
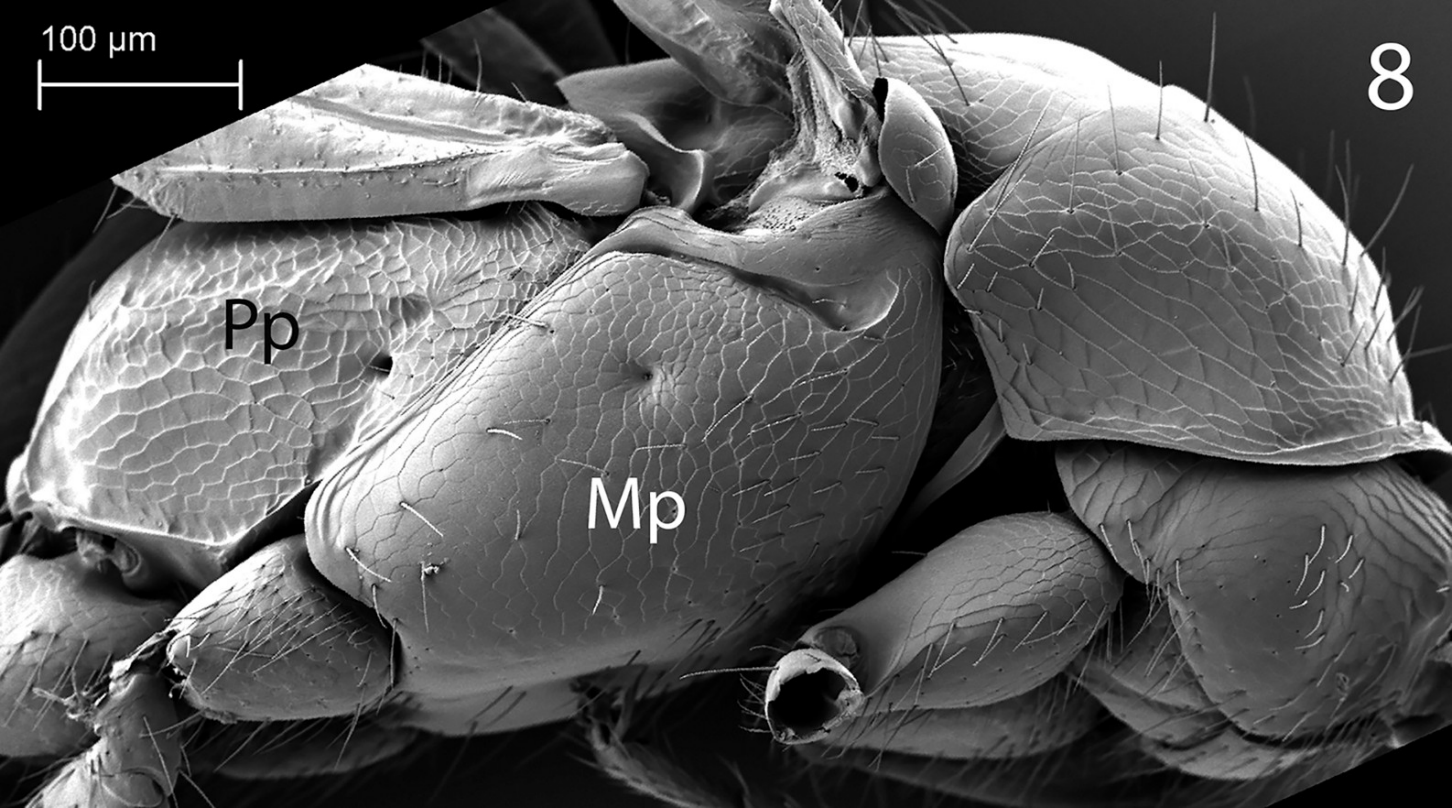
*G*. *omanensis* female paratype, SEM Mesosoma, lateral. Mp = mesopleuron; Pp = propodeum.

**Fig 9:**
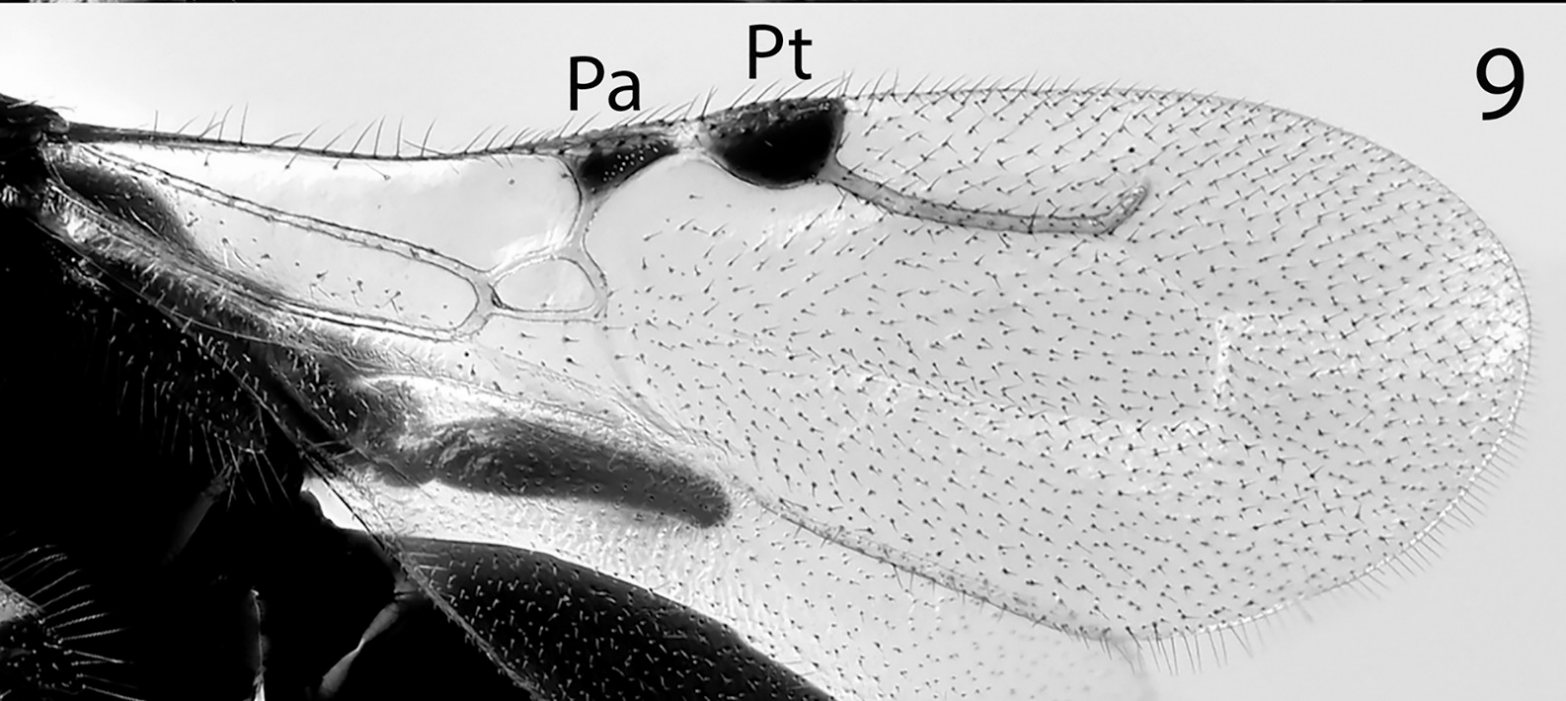
*G*. *omanensis* female paratype, wing. Pa = parastigma; Pt = Pterostigma.

**Fig 10:**
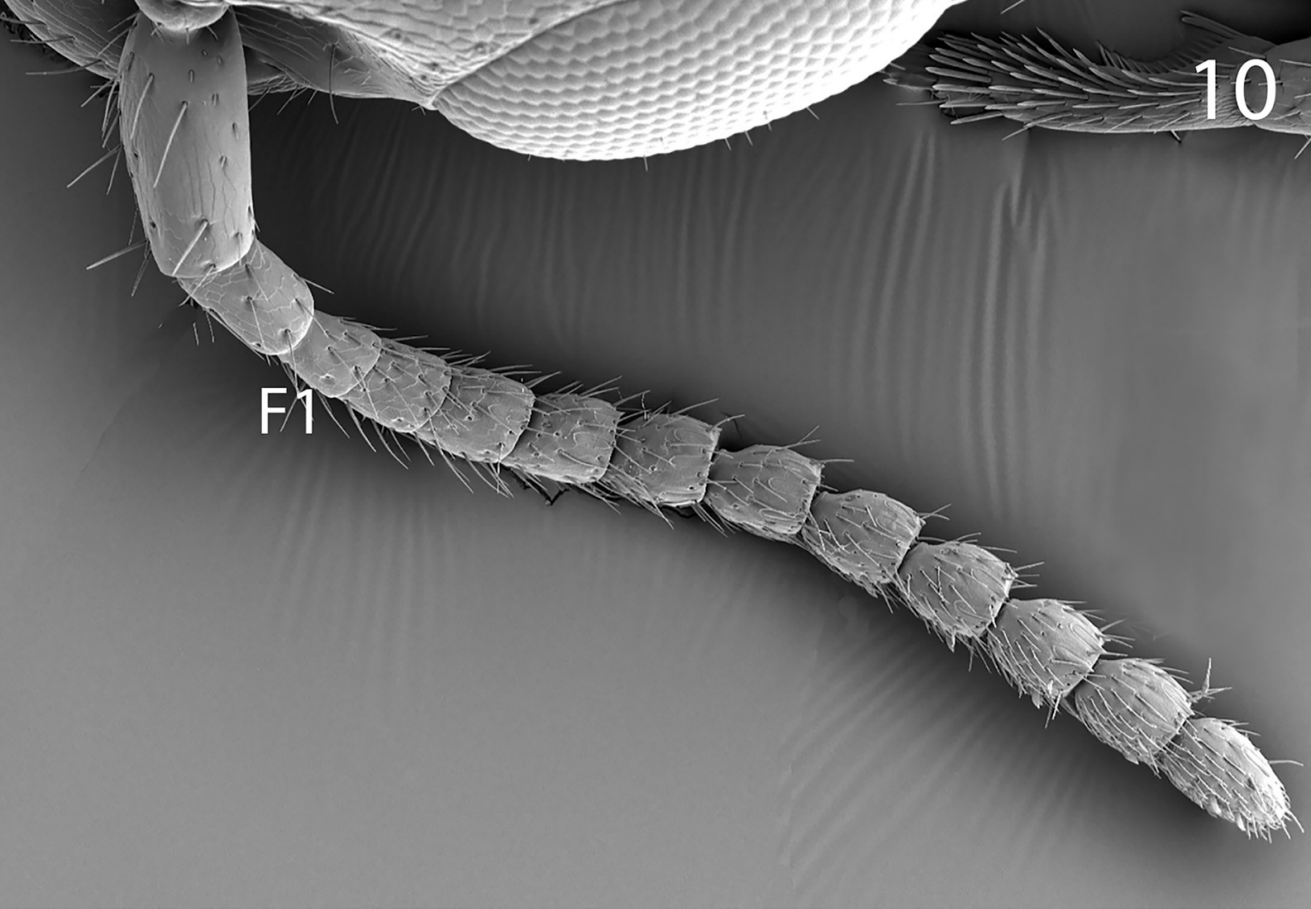
*G*. *omanensis* female paratype, SEM Antenna (female) with F1 indicated.

Female (holotype): length 2.45 mm.

Colour (holotype): almost entirely very dark brown-black, with the following paler (Fig 1): fore femora, distal mid and hind femora and all tarsi, antennae from pedicel to apex; wing spots mid-brown, the same shade as the fore femora; eyes very dark red-maroon; wings hyaline except wing spots and radial vein.

Morphology (holotype): Fore wing length: 1.75 mm. Head 1.2× as long as wide (measured dorsally from centre of dorsal hind margin to clypeal apex and immediately behind eyes). Minimum width of front (frons) 1.56× eye height. Front dull, coriaceous, with setose punctures separated by 3–5 reticulate cells. Ocellar-ocular distance 1.33× width of ocellar triangle (stemmaticum) which is asetose centrally. Each eye with a few (*ca*. 10) short setae. Clypeal margin sinuate. Mandibles with 4 teeth. Antennal segments all either longer than wide or quadrate–none transverse (Figs 1, 2 & 10). Pronotum with reticulate sculpture, the cells larger and smoother than those on head. Posterior pronotum smooth, unsculptured. Anterior mesoscutum smooth, with coarser reticulation posteriorly; anterior setae fine and short, posterior setae as long as those on pronotum, but finer. Parapsidal lines present, stronger posteriorly. Scutellum with very fine setae centrally; two angled grooves anteriorly (Fig 6). Metanotum short, less than 1/20^th^ length of propodeum, with raised reticulation medially. Propodeum with raised reticulation laterally and dorsally at sides, smooth centrally. Mesopleuron (Fig 9) with longitudinal groove (upper fovea) greatly expanded anteriorly, with a deep pit containing a large seta. Petiolar keel present. Metasoma largely smooth with fine reticulate sculpture laterally on T4–T6. Fore wing with 3 closed cells (discoidal cell, “areolet” present), a single row of fine setae across the medial (radial) and submedial (first cubital) cells.

Variation. Length 1.73–2.83 mm. Extensive variation in colour with many specimens, including paratypes with the metasoma lighter than in the holotype. Morphologically extremely uniform.

Male: Morphologically similar to female with the main exception of the genitalia (Figs 2–5). As with many bethylids, the male is relatively easy to distinguish from the female, having the metasoma appearing much less acute distally, while the distal metasoma is more pointed in the female, often with the ovipositor extruded slightly.

**Hosts.** Known so far only from the natural host, *B*. *amydraula*, and the laboratory hosts *Corcyra cephalonica* (Stainton) and *Galleria mellonella* (L.) (Lepidoptera: Pyralidae).

**Distribution.** Iraq, Oman

Material examined: Holotype ♀: OMAN, Barka, Rumais Agriculture and Livestock Research Station 23°29’43.0”N 58°00’ 31.2”E ex *Galleria mellonella* (laboratory host). Original host *Batrachedra amydraula* on *Phoenix dactylifera*, 2006 (same locality). DNA1314: A19 (NHMUK). Paratypes: 6♀ 4♂, same data as holotype (4♀ 2♂, NHMUK; 1♀ 1♂ ONHM, 1♀ 1♂ USNM); 1♂ same data as holotype: DNA1313: A18, genitalia mounted separately on microscope slide (NHMUK). 2♀ same data as holotype, sputter-coated for SEM: DNA1315, DNA1316 (NHMUK).

Other material: IRAQ: unknown locality, 2016; 7♀ 1♂ *ex Batrachedra amydraula* on *Phoenix dactylifera* DNA1312; genitalia of one male mounted on slide (NHMUK).

### Molecular analyses

Out of the six individuals extracted, five yielded usable sequences for both gene fragments in both directions (one specimen from Iraq failed). Sequences have been deposited in GenBank under accession nos MN475307-MN475311 (CO1) and MN476937-MN476941 (28S), respectively.

#### CO1 “barcode” (mitochondrial)

The length of the sequenced CO1 fragment was 814 bp. The Iraq CO1 sequence differs from the Omani sequences at 13 positions of the 814 bp sequence (1.6% uncorrected *p*-distance). These are synonymous C/T and G/A substitutions, as expected form recently diverged sequences. The Iraqi series consists of noticeably larger specimens, apparently bred directly from *B*. *amydraula*, while the Omani specimens were reared on the laboratory host *G*. *mellonella*. Given the morphological and molecular similarities between the two populations we here consider them conspecific, but restrict the type series to Omani specimens because those from Iraq lack exact locality data.

**Fig 11:**
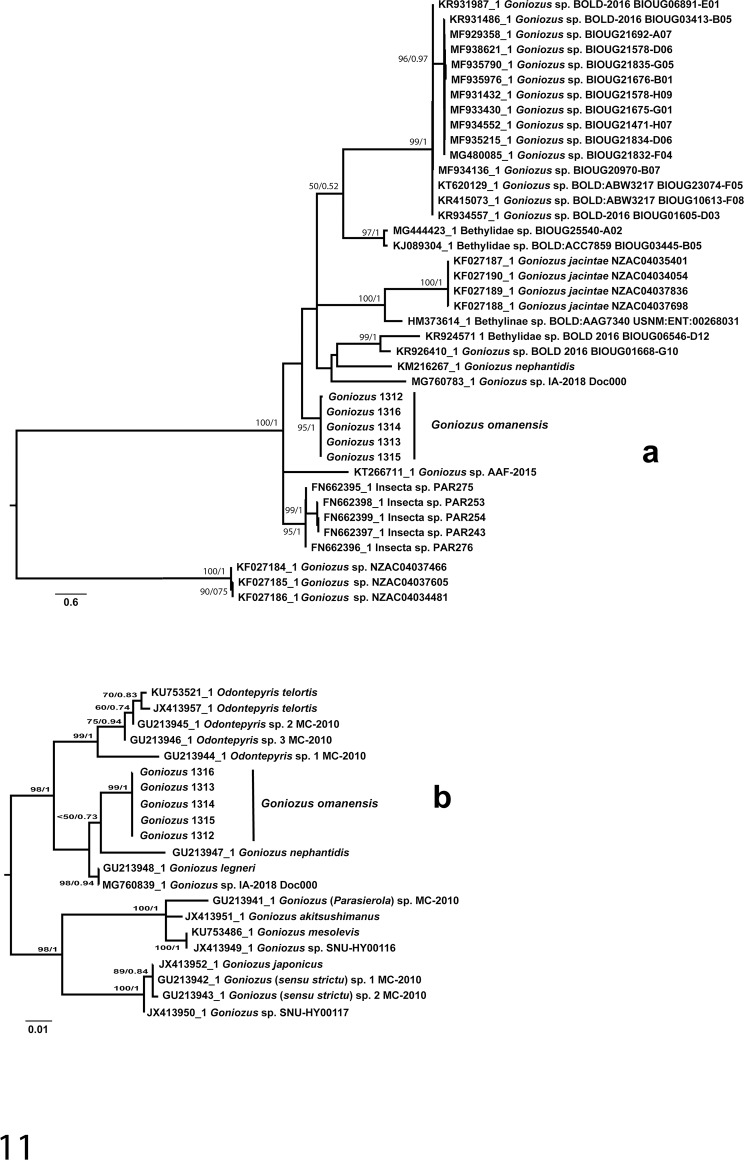
Maximum likelihood trees of *Goniozus* inferred from the analyses of 28S-D2 (a) and CO1 (b) sequences. Likelihood bootstrap support values and posterior probabilities from Bayesian inference analyses above 50 and 0.5, respectively, represented at nodes.

A BLAST of the CO1 Omani sequence into GenBank gave nearest matches of 91.8% with a sequence labelled “Insecta”, with *Goniozus* sp. appearing 7^th^ in the list of closest sequences, with 89.7% similarity. The maximum likelihood phylogenetic analysis (Fig 11A) placed *G*. *omanensis* basal to a clade containing most other available sequences, but with almost no support, while the Bayesian analysis places the species sister to the group of ‘Insecta’ sequences downloaded from GenBank, but again with low support (posterior probability of 0.71; tree not shown).

#### 28S D2 (nuclear ribosomal)

The length of the sequenced 28S D2 fragment was 466 bp. Blasting of the Oman sequence into GenBank gave nearest matches of 97.7% with *G*. *legneri* Gordh and 95.9% with *G*. *nephantidis* (Muesebeck). The maximum likelihood phylogenetic analysis (Fig 11B) placed *G*. *omanensis* as sister to G. *nephantidis*, contrary to the BLAST search that gives higher sequence similarity with *G*. *legneri*. The Bayesian analysis gives the same topology, but with slightly stronger support.

## Discussion

### Taxonomy

There are more than 200 described extant species of *Goniozus* [15] and multiple synonymies among widespread species have been demonstrated [34]. The possibility that *G*. *omanensis* was described previously under another name cannot be ruled out. However, the task of obtaining, or attempting to obtain, each *Goniozus* species holotype to exclude that possible species identity, would at least severely delay, and possibly prohibit, the execution of this work. The morphological and molecular data presented here permit the unequivocal identification of *G*. *omanensis*. Future studies, especially using newly developed technologies, may discover a senior synonym of *G*. *omanensis*.

**Fig 12:**
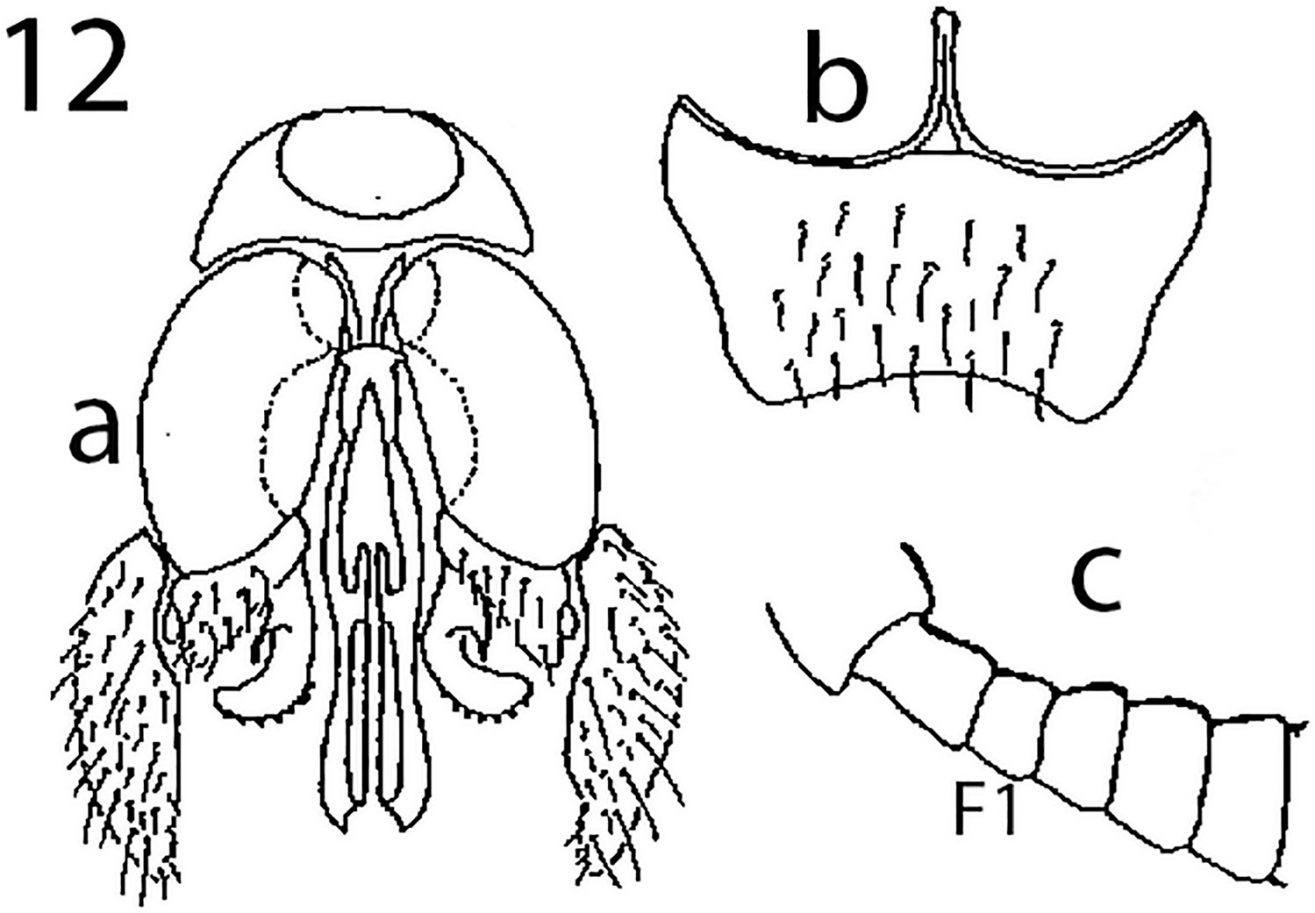
*Goniozus swirskiana* (Argaman). a: ventral genital capsule; b: subgenital plate; c: basal antenna (female) with F1 indicated (all reproduced with permission from Argaman 1992).

Argaman [12] described *Goniozus* (as *Parasierola*) *swirskiana* from the same original host, *B*. *amydraula*, from Israel. Based on the very detailed description, and the original figures reproduced here (Fig 12) it is quite evident that the two species are very distinct (the genitalia figures have been inverted to facilitate comparison with Figs 2, 4 and 5). *G*. *swirskiana* has the female antennal segments F1–F4 transverse (Fig 12C), and Argaman states in the description: “flagellar segments 1–7 transverse”. *G*. *omanensis* has none of the antennal segments transverse (Figs 1, 6 & 10). Equally importantly, the male genitalia of *G*. *swirskiana* (Fig 12A and 12B) are clearly very different from those of *G*. *omanensis* (Figs 2–5). Differences are as follows: the position of the gonocoxae relative to the gonostyIi is appreciably different in the two species, with the outer edges in line in G. *omanensis* and the gonostyli much further away from the centre in *G*. *swirskiana*; the gonobase (“basal ring” of authors) is much narrower in *G*. *swirskiana*, and this character state cannot be explained by squashing or other deformation of the structure during possible slide-mounting. Turning to the subgenital plate, or hypopygium, in *G*. *omanensis* (Fig 2) the plate is approximately rectangular apart from the concave posterior edge, with the spiculum approximately 0.8× the median length of the plate; in *G*. *swirskiana* the plate is rhomboid with the posterior edge much shorter than the anterior edge, and the spiculum just over 0.5× the median length of the plate. We conclude that these three morphological characters unequivocally indicate distinct species status.

**Fig 13:**
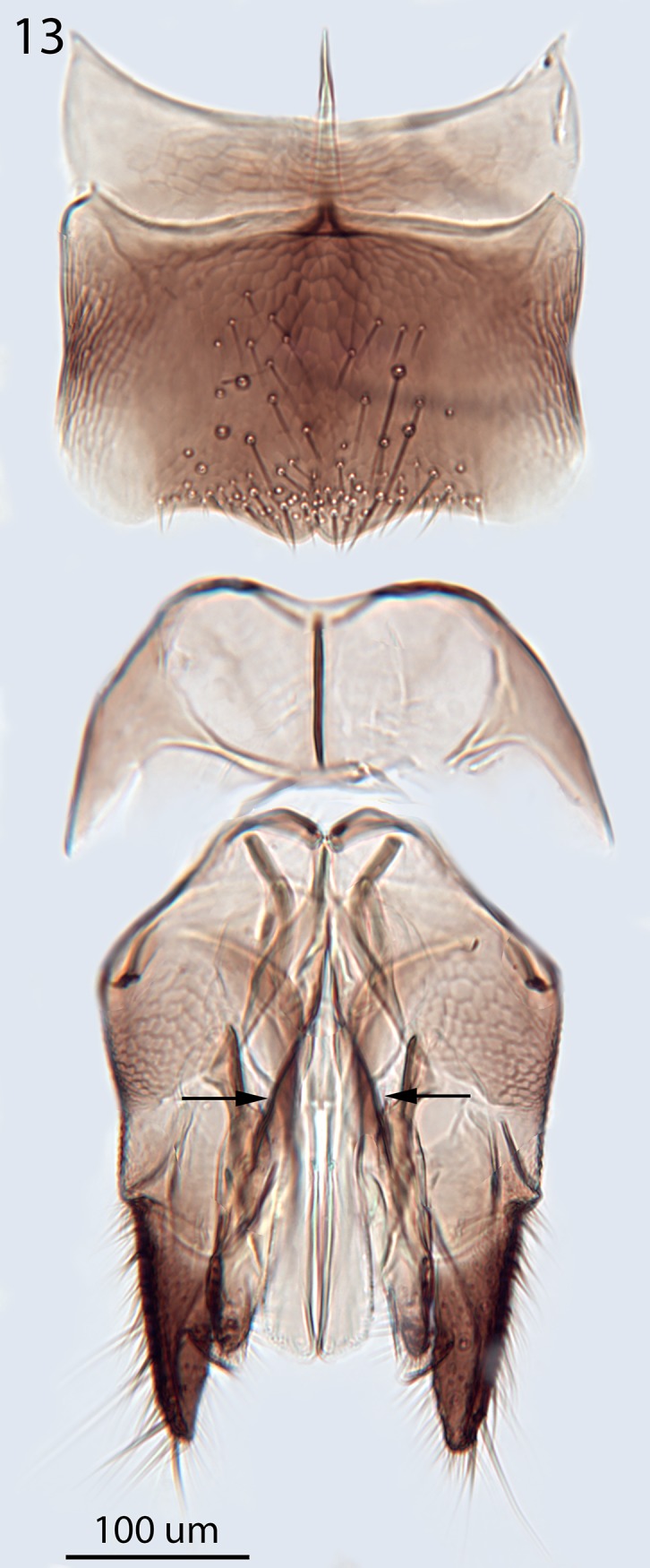
*Goniozus legneri* Gordh. Paratype male, genitalia, subgenital plate + 8^th^ sternite.

**Fig 14:**
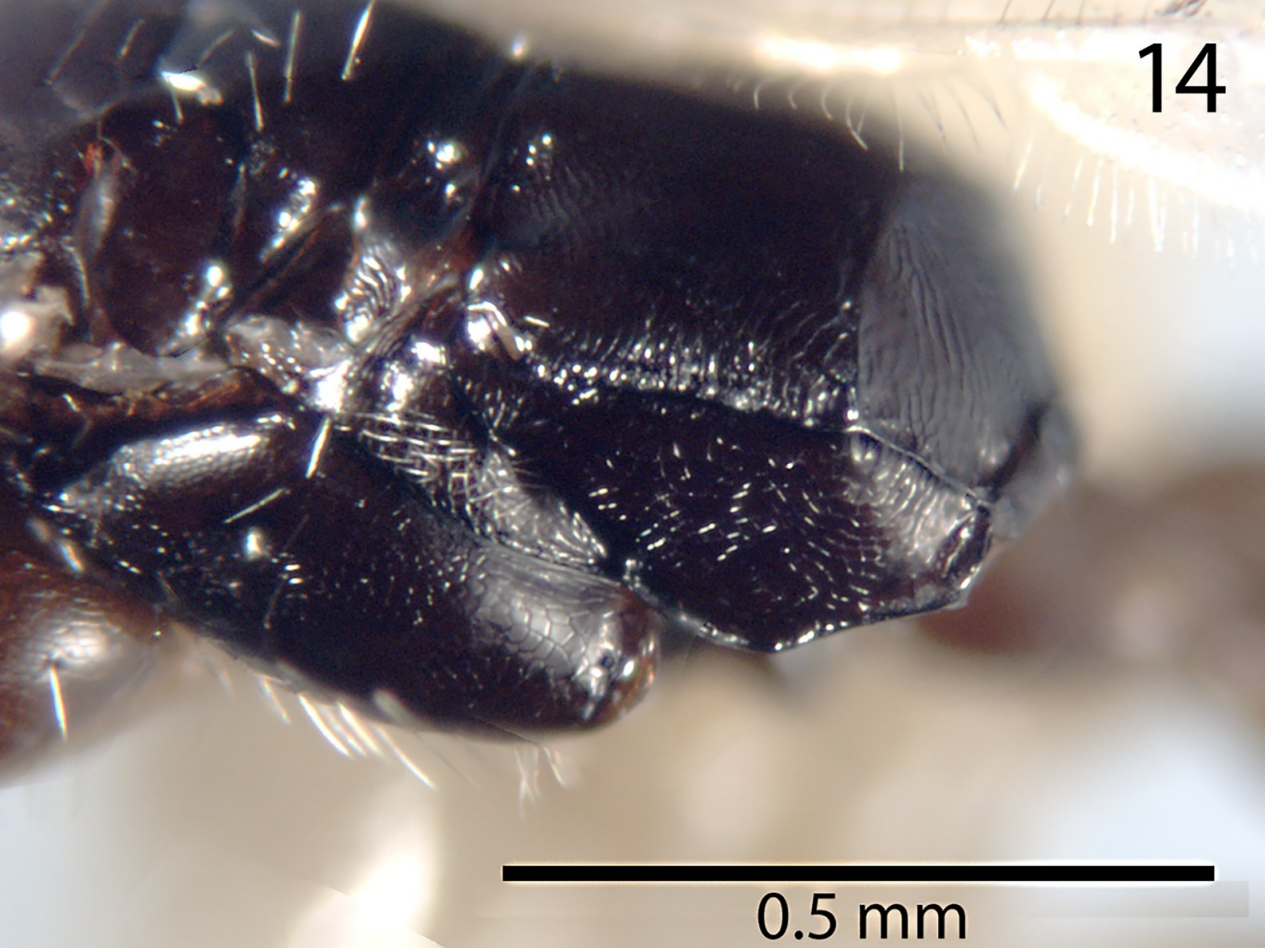
*Goniozus legneri* Gordh. Paratype female, latero-dorsal propodeum.

Because *Goniozus legneri* Gordh appeared to be relatively close to *G*. *omanensis* both in the 28SD2 molecular analysis (see above) and GenBank BLAST, a paratype (NHMUK) of that species was examined, and male genitalia dissected (Fig 13). *G*. *legneri*, originally described from Uruguay, is very readily distinguished from *G*. *omanensis* by a combination of characters, most easily the presence of a transverse posterior propodeal carina (Fig 14). The male genitalia differ in having the penis valves constricted basally (arrowed in Fig 13), and the shape of the subgenital plate is fundamentally different, with the posterior edge convex and bilobed (Fig 13). While *G*. *legneri* might be quite closely related, and was correctly placed by Gordh [35] in the *G*. *punctaticeps* species group of Evans [36], we hesitate here to place *G*. *omanensis* in the same group owing to the major difference in propodeum structure.

Ghahari and Lim [37] recorded *G*. *claripennis* (Foerster) as a parasitoid of *B*. *amydraula* in Iran (Khuzestan). We have not seen the material upon which this record is based, but it could represent a misidentification of *G*. *omanensis*. True *G*. *claripennis* is easily distinguishable from *G*. *omanensis* as it lacks the closed discoidal cell (areolet) in the fore wing.

### Biology and economic importance

*Goniozus omanensis* is a natural enemy of a pest of economic importance, the lesser date moth *Batrachedra amydraula*; as such we here summarize aspects of its biology and its potential as an indigenous agent of biological control in Oman and elsewhere. A video of culture and release of *G*. *omanensis* is available in Supporting Information (S1 Video).

#### Life-history

**Fig 15:**
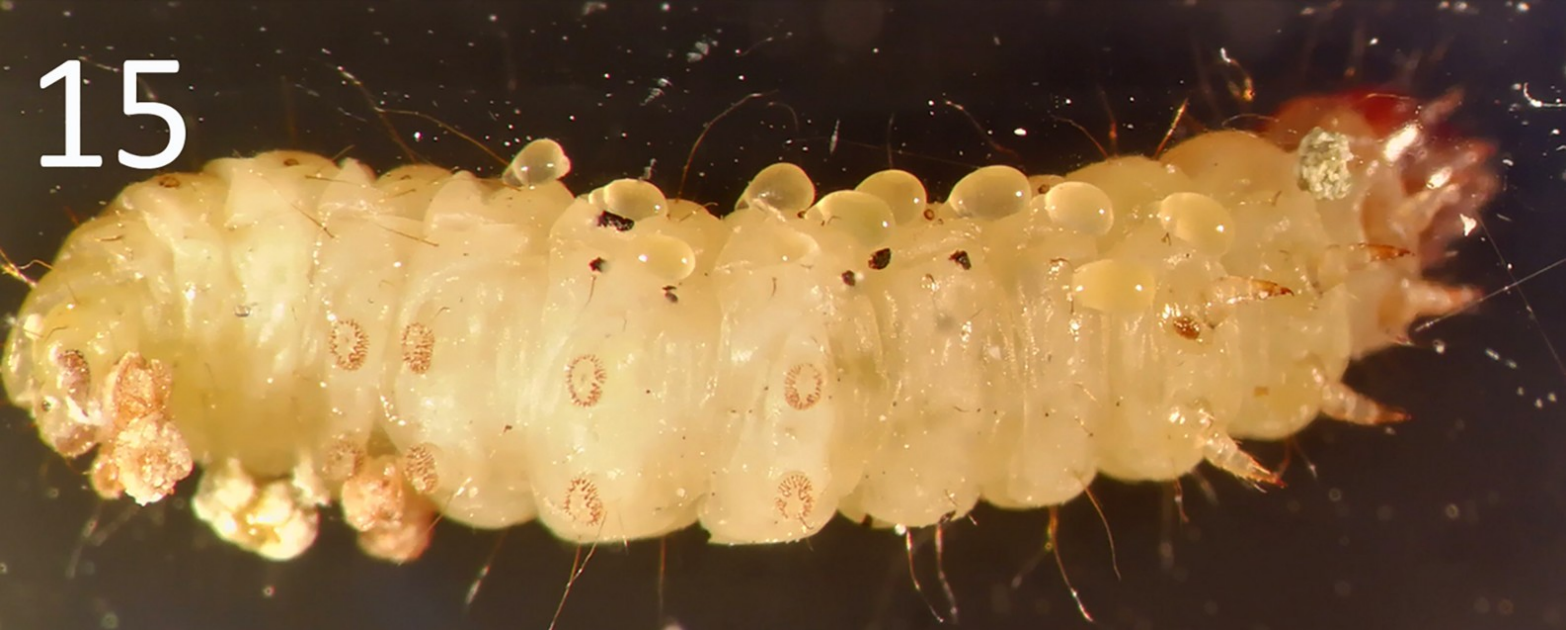
Larvae of *Goniozus omanensis* hatching from eggs laid onto a *Corcyra cephalonica* host larva.

**Fig 16:**
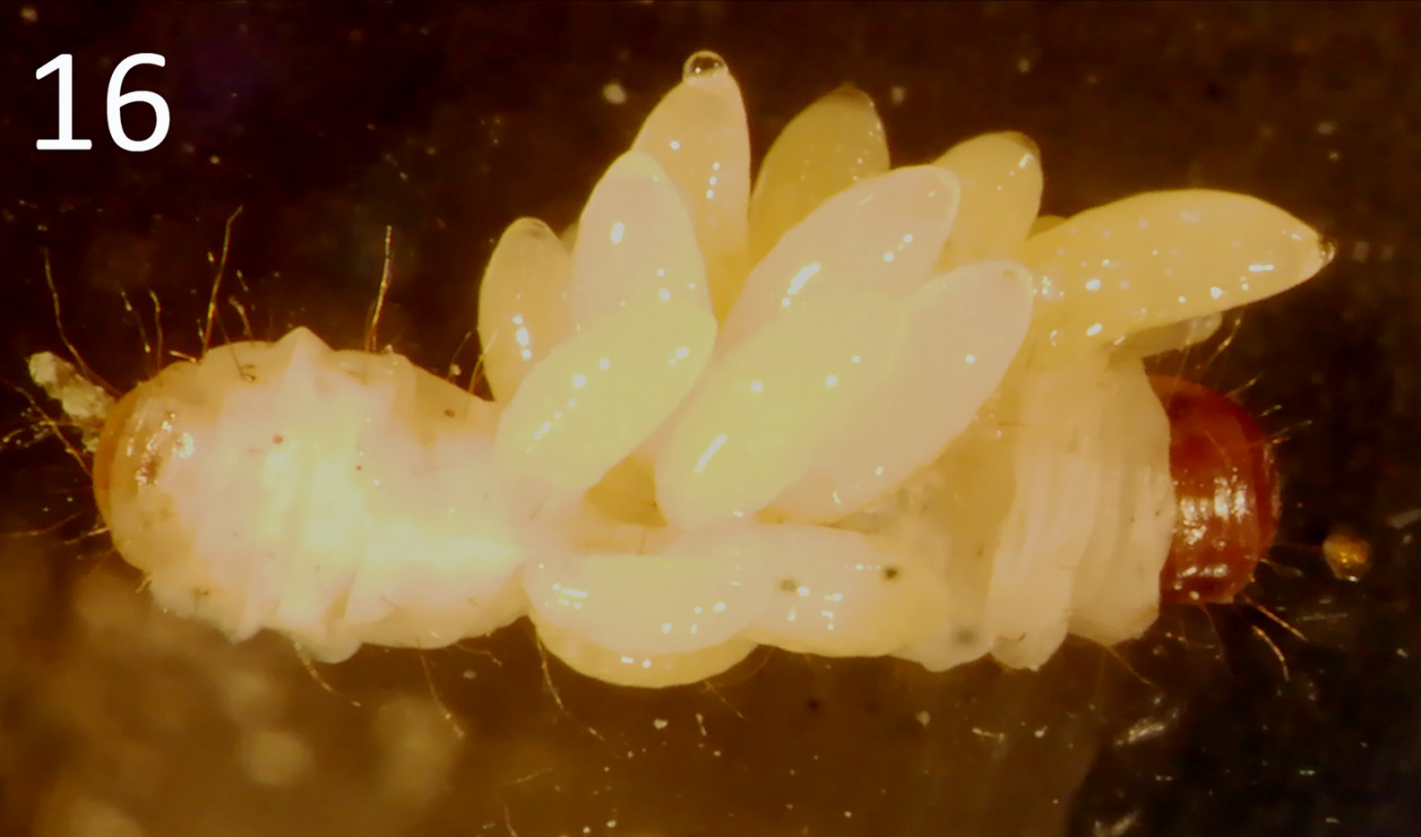
Late-instar larvae of *Goniozus omanensis* feeding on a *Corcyra cephalonica* host larva.

**Fig 17:**
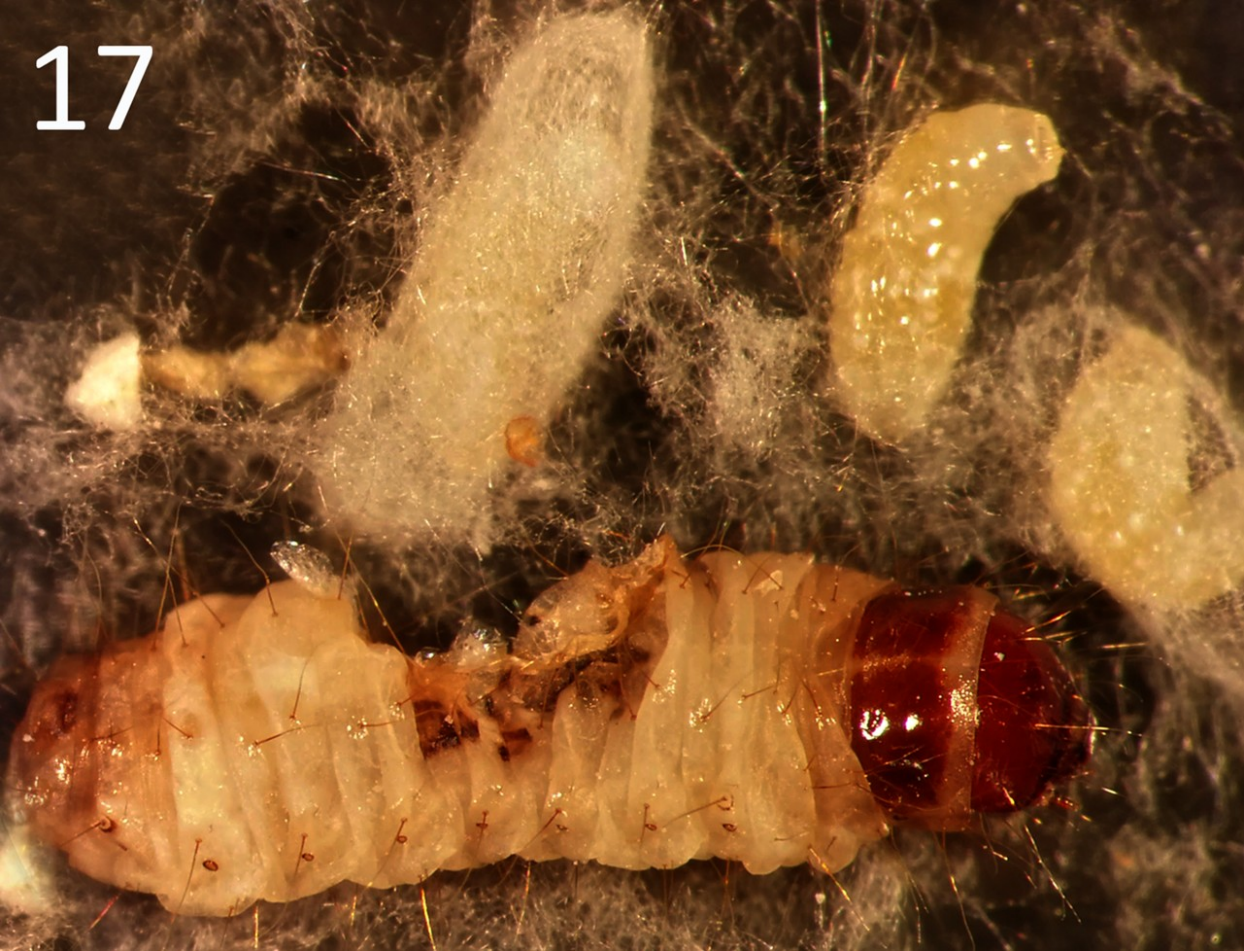
Larvae of *G*. *omanensis* spinning cocoons next to the remains of the host.

The life-history of *G*. *omanensis* is similar to that observed in several congeners [10,38,39,14,40]. After mating (see Supporting Information S2 Video) females attack the larval stage of the host, and paralyse them by stinging, before laying eggs. The eggs hatch after 17–20 hours at 25°C, and newly-hatched larvae begin to feed externally on the host larvae until their growth is completed (3–4 days, Figs 15–17). The parasitoid larvae leave the host and form a white silken cocoon within which they pupate (Fig 17; Supporting Information S3 Video). The pupal stage lasts 7–9 days. The average longevity of the adult male is 3–8 days, and the female 13–33 days (mean 23.5) [9]. When provided with a series of hosts in the laboratory, an individual female can parasitize between 12 and 24 *B*. *amydraula* larvae, and can lay a total of up to 62 eggs during her lifetime [9]. Females may also attack host larvae and feed on them without ovipositing [9]. Offspring sex ratios are typically female biased [9]. Females that have paralysed a host may guard them aggressively against other females (I.H. & S.A.N., pers. obs.), similar to the behaviours reported for congeners [41]. Host and brood guarding behaviours may affect host-parasitoid dynamics, as they constitute a form of mutual interference [40].

Video footage of culture and release, mating behaviour and adult emergence of *G*. *omanensis* is available under Supporting Information (S1 Video, S2 Video, S3 Video).

#### Agro-ecology

A field survey conducted by the Omani Ministry of Agriculture and Fisheries (MAF) on the natural enemies of *B*. *amydraula* in 2006 found six species of parasitoids and two species of predators in infested date fruits [42,9,13,43]. The predators were *Anthocori*s sp. (Hemiptera: Anthocoridae) and *Chrysoperla carnea* (Neuroptera: Chrysopidae) and the hymenopteran parasitoids were *Bracon* sp. (Braconidae), *Apanteles* sp. (Braconidae), *Euderus* near *arenarius* Erdoes (Eulophidae), *Pediobius* sp. (Eulopidae), *Eurytoma* sp. (Eurytomidae) and the *Goniozus* species here described as *G*. *omanensi*s, which was the numerically dominant natural enemy. During the 2007 season, *G*. *omanensi*s was released 2–3 times in 21 date palm farms in five different areas of Oman at a rate of 1–3 females per tree per release [42]. Subsequent surveys of infested fruits found *G*. *omanensi*s more commonly than *B*. *amydraula* (host:parasitoid ratios, April, 1:2.4; May, 1:1.4) whereas in farms without release *B*. *amydraula* was more common (April, 1:0.2; May, 1:0.06) [42]. In farms where *G*. *omanensi*s was released, there were lower levels of pest infestations in the following season [42].

Although these results are promising, the deployment of *G*. *omanensi*s has encountered several challenges and obstacles, including the difficulty of efficiently mass rearing it on its natural host, *B*. *amydraula*. Current work on *G*. *omanensi*s includes the development of techniques to mass rear it in laboratory facilities for augmentative field release. As *B*. *amydraula* larvae enter dormancy (probably diapause) at the end of the third generation (normally in June) and the date fruits are not present in the field on a year-found basis, it is desirable to identify alternative hosts that can be used to rear the parasitoid throughout the year and that are also technically less demanding to maintain in culture than *B*. *amydraula*. Several potential factitious hosts have been tested, including the cotton leafworm *Spodoptera littoralis* (Boisduval) (Lepidoptera: Noctuidae), the greater wax moth *G*. *mellonella*, the Mediterranean flour moth *Ephestia kuehniella* Zeller (Lepidoptera: Pyralidae), and rice moth *C*. *cephalonica* [42]). At present, *G*. *omanensi*s has been successfully reared on *G*. *mellonella* [42] and *C*. *cephalonica* (T.A. unpublished data): both of these host species are relatively straightforward to rear in ventillated jars containing simply prepared diet comprising readily obtained ingredients (glycerol, corn meal, wheat bran, honey and yeast, [44] and are also known to be suitable factitious hosts for some other species of *Goniozus* [39,14,40].

Although *G*. *omanensi*s appears to be the numerically dominant parasitoid in Omani date palm plantations, its population biology may be influenced by behavioural and ecological interactions with other natural enemies of *B*. *amydraula* (as in other agro-ecosystems; [45,46]. We note in particular that there is no information on whether the geographical range of *G*. *omanensi*s, currently recorded from Oman and Iraq, overlaps with those of its congeners *G*. *swirskiana* and *G*. *claripennis*, currently recorded on *B*. *amydraula* in Israel and Iran, respectively [47,48]. Therefore, the occurrence, forms and outcome of any interspecific interactions with congeners, and their consequences for pest population suppression, are unknown.

## Conclusions

We have formally described a new species of bethylid wasp, *Goniozus omanensis* Polaszek. Its biology is similar to that of its congeners and, as a natural enemy of the lesser date moth, *Batrachedra amydraula*, it is a beneficial component of date palm agro-ecosystems in Iraq and Oman. Current studies are aimed at increasing its potential as an agent of biological pest control, especially via the development of efficient mass rearing techniques in order to provide augmentative releases of female parasitoids during the season of pest activity.

## Supporting information

S1 Video *Goniozus omanensis* culture and release. (ZIP)

S2 Video *Goniozus omanensis* mating behaviour. (MP4)

S3 Video *Goniozus omanensis* adults emerging from cocoons. (MP4)
